# Prognostic significance of the preoperative lactate dehydrogenase-to-albumin ratio in patients undergoing radical resection for hilar cholangiocarcinoma

**DOI:** 10.3389/fmed.2025.1705110

**Published:** 2025-11-03

**Authors:** Guoan Li, Tao He, Mingyue Geng, Fuzhen Qi

**Affiliations:** The Affiliated Huaian No. 1 People’s Hospital of Nanjing Medical University, Huaian, China

**Keywords:** hilar cholangiocarcinoma, lactate dehydrogenase-to-albumin ratio, prognostic biomarker, overall survival, radical resection

## Abstract

**Background:**

Hilar cholangiocarcinoma (HCCA) is an aggressive malignancy with a poor prognosis even after curative resection. Accurate prognostic assessment is crucial for individualized treatment and postoperative management. The lactate dehydrogenase-to-albumin ratio (LAR), a composite marker that reflects both tumor metabolism and the host’s nutritional-inflammatory status, has demonstrated prognostic value in several cancers. However, its role in HCCA remains unclear.

**Methods:**

We retrospectively analyzed 112 patients who underwent radical resection for HCCA between 2017 and 2022. Preoperative LAR was calculated from routine laboratory tests. Optimal cut-off values for LAR, neutrophil-to-lymphocyte ratio (NLR), and platelet-to-lymphocyte ratio (PLR) were determined using maximally selected rank statistics. Clinicopathological characteristics were compared between LAR groups. Prognostic factors for overall survival (OS) were evaluated using univariate and multivariate Cox regression analyses. Subgroup analyses assessed the consistency of LAR effects across clinical strata.

**Results:**

The optimal LAR cut-off was 4.67. Patients with high LAR (>4.67) were older and had higher rates of hypertension, lymph node metastasis, and elevated bilirubin, alanine aminotransferase (ALT), and aspartate aminotransferase (AST) levels. In univariate analysis, high LAR, PLR, NLR, carbohydrate antigen 19-9 (CA19-9), total bilirubin (TBIL), ALT, AST, lymph node metastasis, poor differentiation, and R1 resection were significantly associated with worse OS. Multivariate analysis identified high LAR [hazard ratio (HR) 1.70, confidence interval (CI) 1.01–2.87, *p* = 0.046], high PLR (HR 2.12, 95% CI 1.26–3.55, *p* = 0.004), ALT ≥50 U/L (HR 2.94, 95% CI 1.27–6.77, *p* = 0.012), poor differentiation (HR 0.51, 95% CI 0.33–0.83, *p* = 0.006), and microscopically incomplete resection (R1 resection) (HR 2.04, 95% CI 1.14–3.64, *p* = 0.012) as independent predictors. Subgroup analyses showed a consistent adverse effect of high LAR across most strata without significant interactions.

**Conclusion:**

Preoperative LAR is an independent prognostic biomarker for patients with HCCA undergoing radical resection. As a simple, cost-effective, and routinely available index, LAR may assist in risk stratification and postoperative management. External validation is warranted to confirm its clinical utility.

## 1 Introduction

Hilar cholangiocarcinoma (HCCA) is a highly aggressive malignancy that arises at the confluence of the right and left hepatic ducts, accounting for approximately 50–60% of all cholangiocarcinomas (1). HCCA continues to have a poor prognosis, even with advancements in surgical techniques and perioperative management (2). Radical surgical resection offers the sole curative opportunity, yet most patients are diagnosed at an advanced stage and even those undergoing curative resection frequently experience recurrence, leading to unsatisfactory long-term survival outcomes (3). Accurate prognostic assessment is therefore essential for guiding individualized treatment strategies and postoperative surveillance.

In recent years, there has been increasing attention to inflammation- and nutrition-based biomarkers in cancer prognosis. Lactate dehydrogenase (LDH) and albumin (ALB), both routinely measured in clinical practice, reflect distinct aspects of systemic status. LDH, a key glycolytic enzyme, reflects tumor metabolic reprogramming and hypoxia-driven aggressiveness. Elevated LDH levels have been associated with poor prognosis in several cancers (4–6). Conversely, serum albumin is a well-established marker of nutritional status and systemic inflammation, with hypoalbuminemia frequently indicating impaired host immunity and unfavorable outcomes (7, 8). The lactate dehydrogenase-to-albumin ratio (LAR), integrating these two parameters, has gained attention as a novel composite biomarker recently. Growing evidence suggests that LAR possesses prognostic value in multiple malignancies, including esophageal squamous cell carcinoma, gastric cancer, and non-small cell lung cancer (9–11).

However, the value of LAR as a prognostic indicator in HCCA has not yet been fully elucidated. To address this gap, the present study aims to investigate the relationship between preoperative LAR and long-term survival in HCCA patients undergoing surgical resection, and to evaluate its potential as a simple, reliable prognostic indicator for clinical practice.

## 2 Materials and methods

### 2.1 Patients

**Figure 1:**
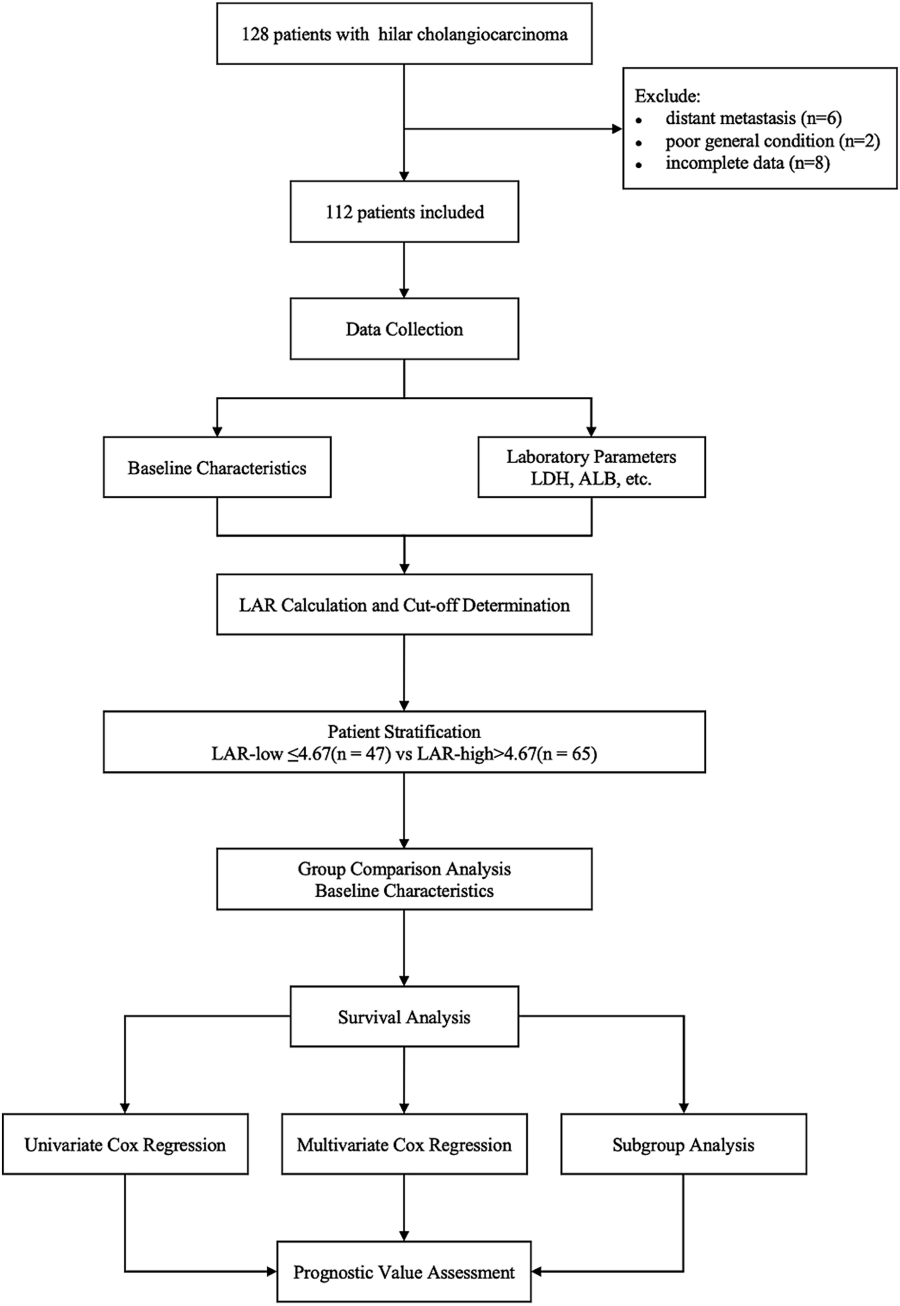
Flow chart of study cohort selection.

Patients diagnosed with hilar cholangiocarcinoma (HCCA) who received radical resection at our hospital between August 2017 and August 2022 were retrospectively analyzed. The research adhered to the Declaration of Helsinki and was approved by the institutional ethics committee (The Affiliated Huaian No. 1 People’s Hospital of Nanjing Medical University, KY-2025-211-01). Given its retrospective design, the requirement for informed consent was waived by the committee. A total of 128 patients with HCCA who received curative-intent surgery were initially identified. Patients were excluded if they had distant metastasis (*n* = 6), poor general condition precluding surgery (*n* = 2), or incomplete clinical data (*n* = 8). After applying these criteria, the final analysis comprised 112 patients (Figure 1).

### 2.2 Data collection

Clinical and pathological data were retrieved from pathological reports and electronic medical records. The variables obtained covered demographic information (body mass index, sex, age) as well as comorbid conditions (hypertension, diabetes), laboratory indicators (CA19-9, total bilirubin, ALT, AST), and tumor characteristics [tumor size, surgical approach (laparoscopic or open), Bismuth–Corlette classification, vascular and perineural invasion, and histological differentiation]. The 8th edition of the American Joint Committee on Cancer (AJCC)/Union for International Cancer Control (UICC) tumor-node-metastasis classification (TNM classification) classification was applied for tumor staging.

### 2.3 Measurement of the LAR index

In the assessment of the LAR index, routine blood parameters measured during the preoperative week were used. The LAR index was calculated as the ratio of serum lactate dehydrogenase (LDH, U/L) to albumin (ALB, g/L), expressed as LAR = LDH/ALB. All laboratory analyses were performed in the central clinical laboratory of our hospital. LDH and Alb levels were measured using the same automated biochemical analyzer with a standardized method throughout the entire study period.

### 2.4 Statistical analysis

We first determined optimal cut-off values for LAR, PLR, and NLR using maximally selected rank statistics (surv_cutpoint in the survminer package) with a minimum proportion per group of 10%. Maximally selected rank statistics is a data-driven method that identifies the threshold that maximizes the difference in survival outcomes between groups. This approach ensures that the selected cut-off values are both statistically significant and clinically meaningful. To further validate these findings and avoid overfitting, we performed cross-validation, where the data was split into multiple subsets to assess the stability and generalizability of the cut-off values. Patients were then categorized accordingly; baseline clinicopathological features were compared between the LAR-low and LAR-high groups with continuous variables analyzed by Student’s *t*-test or Mann–Whitney *U* test and categorical variables assessed by chi-square test or Fisher’s exact test. Clinically accepted thresholds (e.g., CA19-9 37 U/mL, TBIL 34.2 μmol/L, ALT 50 U/L, AST 40 U/L) were used when variables were dichotomized by clinical criteria. Cox proportional hazards models were applied to assess prognostic factors. Factors with *p* < 0.05 in univariable analyses were included in the multivariable model to identify independent predictors. Results are reported as hazard ratios (HRs) with 95% confidence intervals (CIs). Multicollinearity among covariates was examined using the variance inflation factor (VIF), and proportional hazards assumptions were checked using Schoenfeld residuals. Two-sided *p* < 0.05 was considered statistically significant. For subgroup analyses, we estimated the effect of LAR on OS within prespecified strata (e.g., age, sex, diabetes, hypertension, AJCC stage, CA19-9, tumor size, Bismuth–Corlette type, margin status, lymph node status, and tumor differentiation). Potential interactions of LAR with subgroup factors were analyzed through Cox proportional hazards models. Forest plots display subgroup-specific HRs with 95% CIs and *p*-values for interaction. All analyses were performed in R (version 4.2.2; packages: survival, survminer, dplyr, broom, jstable/forestploter, and related utilities).

## 3 Results

### 3.1 Baseline characteristics

**Figure 2:**
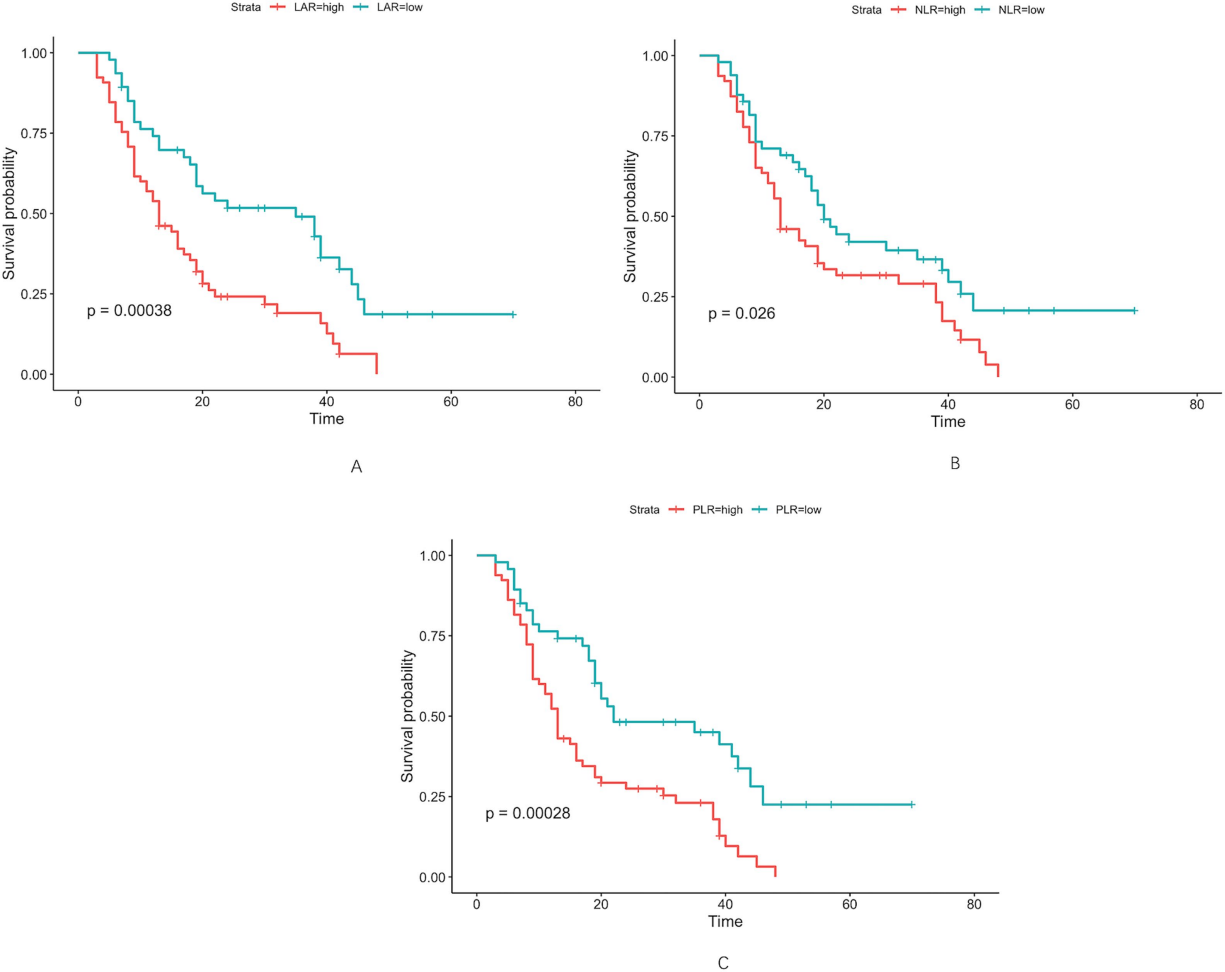
Optimal cut-off values for LAR **(A)**, NLR **(B)**, and PLR **(C)** were determined as 4.67 U/L, 2.98 U/L, and 155.47 U/L, respectively, using maximally selected rank statistics.

Altogether, 112 cases of hilar cholangiocarcinoma met the inclusion criteria and were evaluated. Using maximally selected rank statistics (surv_cutpoint) for overall survival (OS), the optimal cut-off points for LAR, NLR and PLR were 4.67, 2.98 and 155.47, respectively (**Figure** **2**). To evaluate the stability of these cut-off points, internal validation was performed through cross-validation. The coefficient of variation (CV) for LAR, NLR, and PLR was 0.119, 0.125, and 0.092, respectively, indicating good stability and minimal variability across different subsets of the data. For subsequent analyses, patients were stratified into two categories based on LAR values (≤4.67 and >4.67) (Table 1). More patients aged ≥70 years were observed in the LAR-high group (43.08% vs. 17.02%, *p* = 0.004). Gender distribution did not differ significantly (*p* = 0.152). Hypertension was more common in the LAR-high group (50.77% vs. 23.40%, *p* = 0.003), as was lymph node metastasis (36.92% vs. 19.15%, *p* = 0.042). Biochemical variables, such as TBIL (≥34.2 μmol/L), ALT, and AST, showed significant differences, with higher levels in the LAR-high group (TBIL: 86.15% vs. 63.83%, *p* = 0.006). Tumor size and Bismuth–Corlette classification were similar between groups (*p* = 0.665 and *p* = 0.104, respectively).

**Table 1:** Baseline characteristics of hilar cholangiocarcinoma patients.

Variable	Total	Low	High	*p*-value
Age (years)				0.004
<70	76 (67.86%)	39 (82.98%)	37 (56.92%)	
≥70	36 (32.14%)	8 (17.02%)	28 (43.08%)	
Surgical approach				0.696
Open surgery	16 (14.29%)	6 (12.77%)	10 (15.38%)	
Laparoscopy	96 (85.71%)	41 (87.23%)	55 (84.62%)	
Gender				0.152
Male	70 (62.50%)	33 (70.21%)	37 (56.92%)	
Female	42 (37.50%)	14 (29.79%)	28 (43.08%)	
BMI (kg/m^2^)	23.99 ± 3.01	23.55 ± 2.98	24.30 ± 3.01	0.127
Platelet count (×10^9^/L)	225.34 ± 76.65	221.34 ± 91.92	228.23 ± 64.00	0.260
Neutrophils (×10^9^/L)	4.62 ± 1.91	4.39 ± 2.01	4.79 ± 1.83	0.136
Monocytes (×10^9^/L)	0.54 ± 0.24	0.52 ± 0.25	0.56 ± 0.24	0.336
Lymphocytes (×10^9^/l)	1.38 ± 0.50	1.47 ± 0.56	1.32 ± 0.44	0.224
Vascular invasion				0.511
No	73 (65.18%)	29 (61.70%)	44 (67.69%)	
Yes	39 (34.82%)	18 (38.30%)	21 (32.31%)	
Neural invasion				0.942
No	14 (12.50%)	6 (12.77%)	8 (12.31%)	
Yes	98 (87.50%)	41 (87.23%)	57 (87.69%)	
Tumor differentiation				0.370
Poor	46 (41.07%)	17 (36.17%)	29 (44.62%)	
Moderate/Well	66 (58.93%)	30 (63.83%)	36 (55.38%)	
Leakage				0.090
No	92 (82.14%)	42 (89.36%)	50 (76.92%)	
Yes	20 (17.86%)	5 (10.64%)	15 (23.08%)	
Diabetes				0.692
No	97 (86.61%)	40 (85.11%)	57 (87.69%)	
Yes	15 (13.39%)	7 (14.89%)	8 (12.31%)	
Hypertension				0.003
No	68 (60.71%)	36 (76.60%)	32 (49.23%)	
Yes	44 (39.29%)	11 (23.40%)	33 (50.77%)	
Lymph node metastasis				0.042
No	79 (70.54%)	38 (80.85%)	41 (63.08%)	
Yes	33 (29.46%)	9 (19.15%)	24 (36.92%)	
CA19-9 (U/mL)				0.301
≤37	19 (16.96%)	10 (21.28%)	9 (13.85%)	
>37	93 (83.04%)	37 (78.72%)	56 (86.15%)	
Degree of cure				0.418
R0	94 (83.93%)	41 (87.23%)	53 (81.54%)	
R1	18 (16.07%)	6 (12.77%)	12 (18.46%)	
PLR	185.45 ± 97.94	177.77 ± 117.88	191.00 ± 81.09	0.080
NLR	3.87 ± 2.48	3.54 ± 2.67	4.11 ± 2.33	0.050
AJCC stage				0.695
I–II	62 (55.36%)	25 (53.19%)	37 (56.92%)	
III–IV	50 (44.64%)	22 (46.81%)	28 (43.08%)	
TBIL (μmol/L)				0.006
<34.2	26 (23.21%)	17 (36.17%)	9 (13.85%)	
≥34.2	86 (76.79%)	30 (63.83%)	56 (86.15%)	
AST (U/L)				0.094
<40	29 (25.89%)	16 (34.04%)	13 (20.00%)	
≥40	83 (74.11%)	31 (65.96%)	52 (80.00%)	
ALT (U/L)				0.161
<50	26 (23.21%)	14 (29.79%)	12 (18.46%)	
≥50	86 (76.79%)	33 (70.21%)	53 (81.54%)	
Tumor size (cm)				0.665
≤3 cm	88 (78.57%)	36 (76.60%)	52 (80.00%)	
>3 cm	24 (21.43%)	11 (23.40%)	13 (20.00%)	
Bismuth–Corlette				0.104
Type I–II	53 (47.32%)	18 (38.30%)	35 (53.85%)	
Type III–IV	59 (52.68%)	29 (61.70%)	30 (46.15%)	

LAR, lactate dehydrogenase to albumin ratio; CA 19-9, carbohydrate antigen 19-9; NLR, neutrophil-to-lymphocyte ratio; PLR, platelet-to-lymphocyte ratio; BMI, body mass index; ALT, alanine aminotransferase; TBIL, total bilirubin; AST, aspartate aminotransferase; AJCC, American Joint Committee on Cancer.

### 3.2 Univariate and multivariate cox regression analysis

Factors associated with worse survival included high LAR (HR 2.24, 95% CI 1.42–3.53; *p* < 0.001) on univariable analysis (Table 2), lymph node metastasis (HR 2.33, 1.48–3.66; *p* < 0.001), CA19-9 >37 U/mL (HR 1.92, 1.01–3.63; *p* = 0.045), high PLR (HR 2.30, 1.45–3.63; *p* < 0.001), high NLR (HR 1.61, 1.04–2.51; *p* = 0.034), TBIL ≥34.2 μmol/L (HR 2.14, 1.21–3.79; *p* = 0.009), AST ≥40 U/L (HR 1.87, 1.10–3.17; *p* = 0.020), and ALT ≥50 U/L (HR 1.95, 1.12–3.38; *p* = 0.017), whereas a higher lymphocyte count (HR 0.56, 0.35–0.90; *p* = 0.017) and better histological differentiation (moderate/well vs. poor: HR 0.57, 0.37–0.88; *p* = 0.011) were protective; R1 resection was associated with increased risk versus microscopically negative margin (R0) (HR 2.04, 1.17–3.54; *p* = 0.012). Multicollinearity was not detected (all variance inflation factors <2.5; e.g., PLR 1.27, NLR 1.23). In the multivariable model (Table 3), variables with *p* < 0.05 in the univariable analysis were included, and high PLR (HR 2.12, 1.26–3.55; *p* = 0.004), ALT ≥50 U/L (HR 2.94, 1.27–6.77; *p* = 0.012), and high LAR (HR 1.70, 1.01–2.87; *p* = 0.046) remained independent predictors of poorer survival, whereas better differentiation was independently protective (HR 0.51, 0.33–0.83; *p* = 0.006); patients with R1 margins had worse outcomes than those with R0 (HR 2.04, 1.14–3.64; *p* = 0.012). Lymph node metastasis showed a borderline association (HR 1.65, 1.00–2.73; *p* = 0.051), and CA19-9, TBIL, AST, and NLR were not significant in the final model. To assess the predictive performance and stability of the Cox regression model, we performed 10-fold cross-validation. The *C*-index for the model was 0.691, with a standard deviation of 0.114 and a coefficient of variation (CV) of 0.166. The 95% confidence interval for the *C*-index ranged from 0.661 to 0.765. These results demonstrate that the model has good predictive accuracy and stability, effectively distinguishing between risk groups with minimal variability across different data subsets.

**Table 2:** Univariate analyses of OS in hilar cholangiocarcinoma patients.

Variable	HR (95% CI)	*p*-value
Age (<70/≥70 years)	1.01 (0.98–1.03)	0.675
Surgical approach (open surgery/laparoscopy)	1.05 (0.55–1.99)	0.882
Gender (male/female)	1.06 (0.68–1.64)	0.811
BMI (kg/m^2^)	1.02 (0.95–1.09)	0.650
Platelet count (×10^9^/L)	1.00 (1.00–1.00)	0.135
Neutrophils (×10^9^/L)	1.05 (0.93–1.18)	0.425
Monocytes (×10^9^/L)	1.01 (0.43–2.40)	0.979
Lymphocytes (×10^9^/L)	0.56 (0.35–0.90)	0.017
Vascular invasion (no/yes)	1.13 (0.72–1.75)	0.595
Perineural invasion (no/yes)	1.06 (0.56–1.99)	0.867
Bile leakage (no/yes)	1.09 (0.63–1.88)	0.759
Diabetes (no/yes)	0.57 (0.26–1.24)	0.157
Hypertension (no/yes)	0.76 (0.48–1.19)	0.232
Lymph node metastasis (no/yes)	2.33 (1.48–3.66)	<0.001
CA19-9 (≤37/>37 U/mL)	1.92 (1.01–3.63)	0.045
Degree of cure (R0/R1)	2.04 (1.17–3.54)	0.012
NLR (low/high)	1.61 (1.04–2.51)	0.034
PLR (low/high)	2.30 (1.45–3.63)	<0.001
TNM stage (I–II/III–IV)	0.88 (0.57–1.36)	0.575
Total bilirubin (<34.2/≥34.2 μmol/L)	2.14 (1.21–3.79)	0.009
AST (<40/≥40 U/L)	1.87 (1.10–3.17)	0.020
ALT (<50/≥50 U/L)	1.95 (1.12–3.38)	0.017
Tumor size (≤3/>3 cm)	1.13 (0.66–1.93)	0.652
Bismuth–Corlette (type I–II/type III–IV)	0.76 (0.49–1.16)	0.199
LAR (low/high)	2.24 (1.42–3.53)	<0.001
AJCC stage (poor/moderate and well)	0.57 (0.37–0.88)	0.011

LAR, lactate dehydrogenase to albumin ratio; CA 19-9, carbohydrate antigen 19-9; NLR, neutrophil-to-lymphocyte ratio; PLR, platelet-to-lymphocyte ratio; BMI, body mass index; ALT, alanine aminotransferase; TBIL, total bilirubin; AST, aspartate aminotransferase; AJCC, American Joint Committee on Cancer.

**Table 3:** Multivariate analyses of OS in hilar cholangiocarcinoma patients.

Variable	HR (95% CI)	*p*-value
Lymph node metastasis (no/yes)	1.65 (1.00–2.73)	0.051
CA19-9 (≤37/>37 U/mL)	1.35 (0.62–2.97)	0.449
Degree of cure (R0/R1)	2.04 (1.14–3.64)	0.012
NLR (low/high)	0.89 (0.55–1.46)	0.653
PLR (low/high)	2.12 (1.26–3.55)	0.004
Total bilirubin (<34.2/≥34.2 μmol/L)	1.10 (0.57–2.15)	0.773
AST (<40/≥40 U/L)	0.54 (0.24–1.23)	0.143
ALT (<50/≥50 U/L)	2.94 (1.27–6.77)	0.012
LAR (no/high)	1.70 (1.01–2.87)	0.046
AJCC stage (poor/moderate and well)	0.51 (0.33–0.83)	0.006

LAR, lactate dehydrogenase to albumin ratio; CA 19-9, carbohydrate antigen 19-9; NLR, neutrophil-to-lymphocyte ratio; PLR, platelet-to-lymphocyte ratio; ALT, alanine aminotransferase; TBIL, total bilirubin; AST, aspartate aminotransferase; AJCC, American Joint Committee on Cancer.

### 3.3 Subgroup analysis

**Figure 3:**
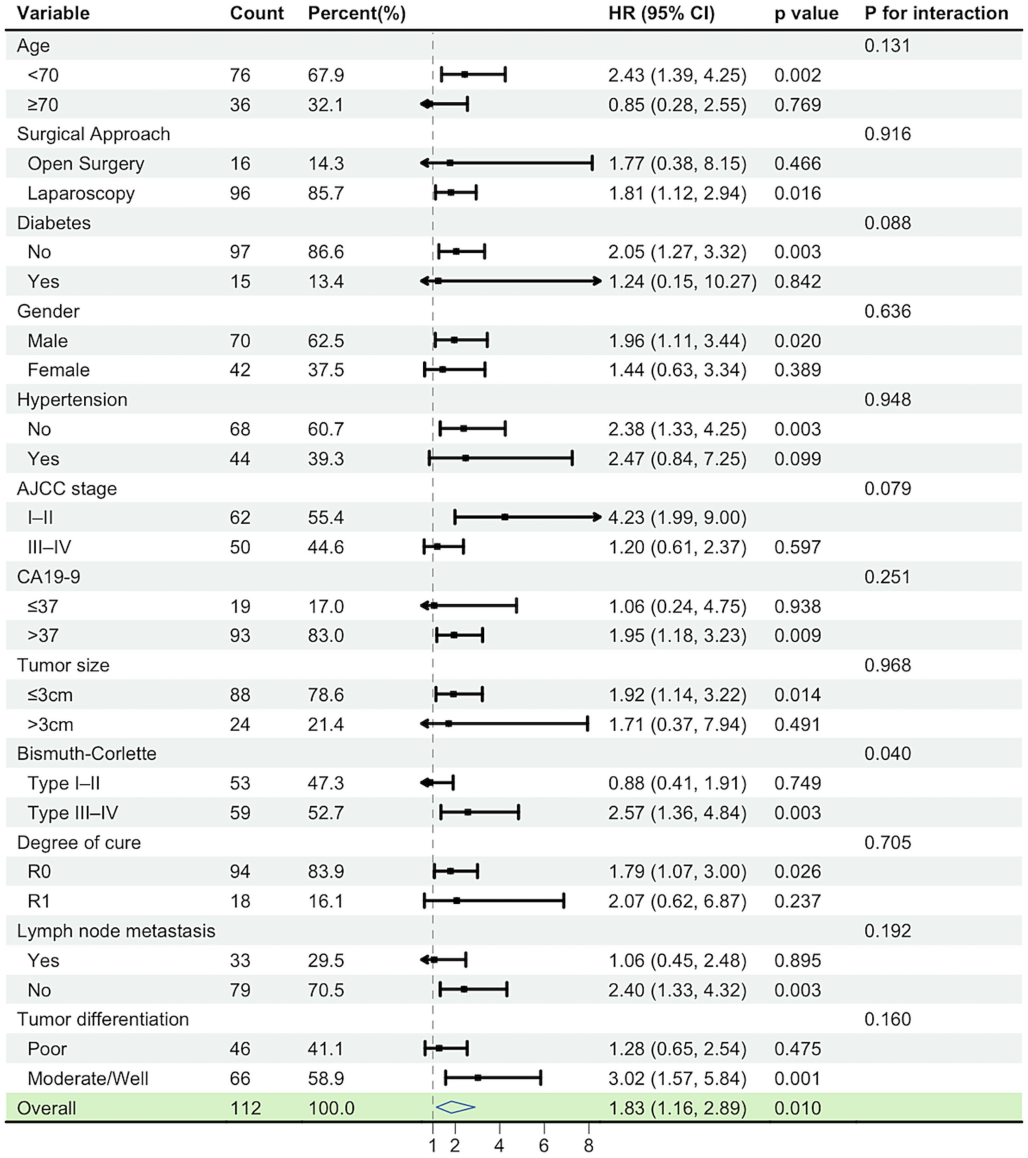
Subgroup analyses of overall survival according to preoperative LAR status.

In the subgroup forest plot, the association between high LAR and worse overall survival (OS) was directionally consistent across most prespecified strata (HRs predominantly >1), with 95% CIs crossing unity in only a few subgroups. No significant interactions were detected (all *p* for interaction >0.05), indicating a robust and consistent adverse effect of high LAR across clinical subgroups. Point estimates tended to be larger in higher-risk strata (e.g., R1 margins, stage III–IV, node-positive), but there was no statistical evidence of effect modification. These findings align with the multivariable Cox model in which LAR remained an independent adverse prognostic factor. The model was adjusted for PLR, AJCC stage (TNM), surgical margin, and ALT levels (Figure 3).

## 4 Discussion

In this study, we identified the preoperative LAR as an independent prognostic factor of survival for patients with HCCA undergoing radical resection. Individuals in the high-LAR group had notably worse outcomes than those in the low-LAR group, and the prognostic impact of LAR remained significant after adjusting for other clinicopathological factors. These results indicate that LAR, a simple and inexpensive index derived from routine laboratory tests, could serve as a valuable marker for risk stratification.

Elevated LDH reflects a Warburg phenotype in which cancer cells convert pyruvate to lactate despite oxygen availability, regenerating NAD^+^ to sustain high glycolytic flux and the biosynthetic and energetic needs of rapid proliferation (12). This metabolic shift not only fuels tumor growth but also promotes invasive behavior and resistance to cell death (13). LDH has been shown to correlate with tumor progression, metastasis, and poor prognosis in various cancers, including biliary tract cancer (14). By measuring LDH levels, we indirectly assess the metabolic aggressiveness of the tumor, which is critical for predicting survival outcomes in patients undergoing curative resection. Conversely, hypoalbuminemia serves as an indicator of malnutrition and systemic inflammation (8). Low serum albumin levels often result from chronic inflammation, which is a hallmark of many cancers. Inflammatory cytokines, notably tumor necrosis factor-alpha (TNF-α) and interleukin-6 (IL-6), can induce the liver to decrease albumin production, while simultaneously enhancing the synthesis of acute-phase proteins (15). The presence of systemic inflammation impairs immune function, disrupts normal tissue repair mechanisms, and enhances cancer cell survival, thereby contributing to cancer progression (16, 17). Additionally, hypoalbuminemia is a well-established indicator of poor nutritional status, which is frequently observed in cancer patients due to decreased intake, malabsorption, and catabolic states driven by the tumor (18, 19). By combining LDH and albumin, the LAR simultaneously captures tumor biology and the host’s systemic inflammatory-nutritional response, making it a more robust, multifaceted biomarker.

In line with our findings in HCCA, recent studies in other tumor types have demonstrated that the LDH-to-albumin ratio (LAR) is a significant prognostic biomarker. For example, in extranodal NK/T-cell lymphoma, higher LAR predicted worse OS and progression-free survival (PFS) after controlling for baseline features (20). In oral cancer, high preoperative LAR was recognized as an independent prognostic factor of poor overall survival (21). Similarly, in breast cancer, patients with elevated preoperative LAR exhibited worse progression-free survival compared with the low-LAR group (22). High preoperative LAR in bladder cancer was also linked to worse overall and recurrence-free survival outcomes (23). In our cohort, high PLR was an independent predictor of poorer survival, consistent with the findings of Saito et al. (24), who reported that elevated PLR demonstrated a significant relationship with reduced survival. In contrast, Lin et al. (25) reported that LMR, rather than PLR or NLR, was the most robust prognostic marker, suggesting variability across cohorts and analytic strategies. Our study did not identify NLR as an independent prognostic factor, in contrast to Dumitrascu et al. (26), who demonstrated its significance together with R0 resection and adjuvant therapy. Such inconsistency may be attributable to variations in cut-off definitions, cohort size, or therapeutic strategies.

This study has several limitations that should be considered. First, the single-center, retrospective design, and relatively small sample size may limit the generalizability of our findings and introduce potential selection bias. The relatively small cohort is partly due to the rarity of HCCA, a rare and highly aggressive malignancy, which naturally restricts the number of eligible patients. Additionally, the strict inclusion and exclusion criteria, which aimed to select a homogeneous patient group for more reliable analysis, further contributed to the smaller cohort size. Therefore, the sample size reflects both the rarity of the disease and the predefined selection criteria. While the model is encouraging, its predictive accuracy as measured by the *C*-index (0.691) still has room for improvement, and the small sample size may limit the statistical power of some subgroup analyses. External validation with larger, multicenter cohorts is needed to confirm the robustness and applicability of our findings. Secondly, the study did not include details regarding postoperative management, such as adjuvant therapy, recurrence status, or complications, which could potentially affect long-term outcomes. These factors, if incorporated into future studies, may offer a more comprehensive view of the prognostic significance of LAR. Furthermore, dynamic changes in LAR over time were not analyzed in this study. Given that LAR is a marker that could fluctuate in response to tumor progression or treatment, future research incorporating serial measurements of LAR during the perioperative or adjuvant treatment periods could provide additional prognostic insights. This would enhance our understanding of how LAR reflects the ongoing tumor biology and the host’s systemic inflammatory-nutritional response.

## 5 Conclusion

Preoperative lactate dehydrogenase-to-albumin ratio (LAR) is an independent prognostic factor in patients with hilar cholangiocarcinoma after radical resection. As a simple and inexpensive biomarker reflecting tumor metabolism and host status, LAR may assist in risk stratification and postoperative management. Further multicenter prospective studies are needed to validate its prognostic utility.

## Data Availability

The original contributions presented in the study are included in the article/supplementary material, further inquiries can be directed to the corresponding author.

## Glossary

HCCA: Hilar cholangiocarcinoma
LAR: Lactate dehydrogenase-to-albumin ratio
LDH: Lactate dehydrogenase
ALB: Albumin
NLR: Neutrophil-to-lymphocyte ratio
PLR: Platelet-to-lymphocyte ratio
OS: Overall survival
HR: Hazard ratio
CI: Confidence interval
TBIL: Total bilirubin
ALT: Alanine aminotransferase
AST: Aspartate aminotransferase
CA19-9: Carbohydrate antigen 19-9
AJCC: American Joint Committee on Cancer
UICC: Union for International Cancer Control
VIF: Variance inflation factor
R0/R1: Resection margin status
PFS: Progression-free survival
*p*: *p*-value
TNM: Tumor-node-metastasis classification

## References

[1] Banales JM Marin JJG Lamarca A Rodrigues PM Khan SA Roberts LR. Cholangiocarcinoma 2020: the next horizon in mechanisms and management. Nat Rev Gastroenterol Hepatol. (2020) 17:557–88. doi: 10.1038/s41575-020-0310-z, PMID: 32606456 PMC7447603

[2] Lee SH Choi GH Han DH Kim KS Choi JS Rho SY. Chronological analysis of surgical and oncological outcomes after the treatment of perihilar cholangiocarcinoma. Ann Hepatobiliary Pancreat Surg. (2021) 25:62–70. doi: 10.14701/ahbps.2021.25.1.62, PMID: 33649256 PMC7952679

[3] Quinn LM Dunne DFJ Jones RP Poston GJ Malik HZ Fenwick SW. Optimal perioperative care in peri-hilar cholangiocarcinoma resection. Eur Surg. (2018) 50:93–9. doi: 10.1007/s10353-018-0529-x, PMID: 29875797 PMC5968056

[4] Wu M Lin P Xu L Yu Z Chen Q Gu H. Prognostic role of serum lactate dehydrogenase in patients with urothelial carcinoma: a systematic review and Meta-analysis. Front Oncol. (2020) 10:677. doi: 10.3389/fonc.2020.00677, PMID: 32509573 PMC7252225

[5] Li Y Wang K Zhao E Li B Li S Dong X. Prognostic value of lactate dehydrogenase in second-line immunotherapy for advanced esophageal squamous cell carcinoma. Pathol Oncol Res. (2022) 28:1610245. doi: 10.3389/pore.2022.1610245, PMID: 35721326 PMC9203685

[6] Forkasiewicz A Dorociak M Stach K Szelachowski P Tabola R Augoff K. The usefulness of lactate dehydrogenase measurements in current oncological practice. Cell Mol Biol Lett. (2020) 25:35. doi: 10.1186/s11658-020-00228-7, PMID: 32528540 PMC7285607

[7] Almasaudi AS Dolan RD Edwards CA McMillan DC. Hypoalbuminemia reflects nutritional risk, body composition and systemic inflammation and is independently associated with survival in patients with colorectal Cancer. Cancer. (2020) 12:1986. doi: 10.3390/cancers12071986, PMID: 32708140 PMC7409314

[8] Andreescu J Ocanto A Couñago F. Prognostic significance of nutritional and inflammatory markers in colorectal cancer. World J Clin Oncol. (2025) 16:104958. doi: 10.5306/wjco.v16.i6.104958, PMID: 40585821 PMC12198870

[9] Luo M Wei H Qiu M Su C Ning R Zhou S. Prognostic value of the lactate dehydrogenase to albumin ratio in advanced non-small cell lung cancer patients treated with the first-line PD-1 checkpoint inhibitors combined with chemotherapy. Front Immunol. (2025) 16:1473962. doi: 10.3389/fimmu.2025.1473962, PMID: 40013138 PMC11861202

[10] Aday U Tatlı F Akpulat FV İnan M Kafadar MT Bilge H. Prognostic significance of pretreatment serum lactate dehydrogenase-to-albumin ratio in gastric cancer. Contemp Oncol. (2020) 24:145–9. doi: 10.5114/wo.2020.100219, PMID: 33235539 PMC7670180

[11] Feng JF Wang L Yang X Jiang YH. Prognostic value of lactate dehydrogenase to albumin ratio (LAR) in patients with resectable esophageal squamous cell carcinoma. Cancer Manag Res. (2019) 11:7243–51. doi: 10.2147/CMAR.S208320, PMID: 31447584 PMC6683178

[12] Liberti MV Locasale JW. The Warburg effect: How does it benefit cancer cells? Trends Biochem Sci. (2016) 41:211–8. doi: 10.1016/j.tibs.2015.12.001, PMID: 26778478 PMC4783224

[13] Zhong X He X Wang Y Hu Z Huang H Zhao S. Warburg effect in colorectal cancer: the emerging roles in tumor microenvironment and therapeutic implications. J Hematol Oncol. (2022) 15:160. doi: 10.1186/s13045-022-01358-5, PMID: 36319992 PMC9628128

[14] Ma L Qiu J Zhang Y Qiu T Wang B Chen W. Prognostic factors for operable biliary tract cancer: serum levels of lactate dehydrogenase, a strong association with survival. Onco Targets Ther. (2018) 11:2533–43. doi: 10.2147/OTT.S150502, PMID: 29765232 PMC5942178

[15] Mantovani A Garlanda C. Humoral innate immunity and acute-phase proteins. N Engl J Med. (2023) 388:439–52. doi: 10.1056/NEJMra2206346, PMID: 36724330 PMC9912245

[16] Shalapour S Karin M. Immunity, inflammation, and cancer: an eternal fight between good and evil. J Clin Invest. (2015) 125:3347–55. doi: 10.1172/JCI80007, PMID: 26325032 PMC4588298

[17] Yu H Pardoll D Jove R. STATs in cancer inflammation and immunity: a leading role for STAT3. Nat Rev Cancer. (2009) 9:798–809. doi: 10.1038/nrc2734, PMID: 19851315 PMC4856025

[18] Fearon K Strasser F Anker SD Bosaeus I Bruera E Fainsinger RL. Definition and classification of cancer cachexia: an international consensus. Lancet Oncol. (2011) 12:489–95. doi: 10.1016/S1470-2045(10)70218-7, PMID: 21296615

[19] Guven DC Sahin TK Erul E Rizzo A Ricci AD Aksoy S. The association between albumin levels and survival in patients treated with immune checkpoint inhibitors: a systematic review and meta-analysis. Front Mol Biosci. (2022) 9:1039121. doi: 10.3389/fmolb.2022.1039121, PMID: 36533070 PMC9756377

[20] Li N Feng Y Chen X Zou LQ. The prognostic value of lactate dehydrogenase/albumin ratio in extranodal natural killer/T cell lymphoma. BMC Cancer. (2025) 25:1176. doi: 10.1186/s12885-025-14393-5, PMID: 40665221 PMC12261561

[21] Wang X Ji X. Effect of preoperative serum lactate dehydrogenase-to-albumin ratio on the survival of oral cancer: a retrospective study. J Inflamm Res. (2024) 17:5129–38. doi: 10.2147/JIR.S472041, PMID: 39104906 PMC11298564

[22] He J Tong L Wu P Wu Y Shi W Chen L. Prognostic significance of preoperative lactate dehydrogenase to albumin ratio in breast cancer: a retrospective study. Int J Gen Med. (2023) 16:507–14. doi: 10.2147/IJGM.S396871, PMID: 36789133 PMC9922482

[23] Xu H Lin T Ai J Zhang J Zhang S Li Y. Utilizing the lactate dehydrogenase-to-albumin ratio for survival prediction in patients with bladder cancer after radical cystectomy. J Inflamm Res. (2023) 16:1733–44. doi: 10.2147/JIR.S384338, PMID: 37096127 PMC10122464

[24] Saito H Noji T Okamura K Tsuchikawa T Shichinohe T Hirano S. A new prognostic scoring system using factors available preoperatively to predict survival after operative resection of perihilar cholangiocarcinoma. Surgery. (2016) 159:842–51. doi: 10.1016/j.surg.2015.10.027, PMID: 26683498

[25] Lin ZQ Ma C Cao WZ Ning Z Tan G. Prognostic significance of NLR, PLR, LMR and tumor infiltrating T lymphocytes in patients undergoing surgical resection for hilar cholangiocarcinoma. Front Oncol. (2022) 12:908907. doi: 10.3389/fonc.2022.908907, PMID: 35719959 PMC9203898

[26] Dumitrascu T Chirita D Ionescu M Popescu I. Resection for hilar cholangiocarcinoma: analysis of prognostic factors and the impact of systemic inflammation on long-term outcome. J Gastrointest Surg. (2013) 17:913–24. doi: 10.1007/s11605-013-2144-2, PMID: 23319395

